# Fungal Community as a Bioindicator to Reflect Anthropogenic Activities in a River Ecosystem

**DOI:** 10.3389/fmicb.2018.03152

**Published:** 2018-12-21

**Authors:** Yaohui Bai, Qiaojuan Wang, Kailingli Liao, Zhiyu Jian, Chen Zhao, Jiuhui Qu

**Affiliations:** ^1^Key Laboratory of Drinking Water Science and Technology, Research Center for Eco-Environmental Sciences, Chinese Academy of Sciences, Beijing, China; ^2^University of Chinese Academy of Sciences, Beijing, China

**Keywords:** anthropogenic activity, fungal community, mycoplankton, biofilm, bioindicator

## Abstract

The fungal community interacts with the ambient environment and can be used as a bioindicator to reflect anthropogenic activities in aquatic ecosystems. Several studies have investigated the impact of anthropogenic activities on the fungal community and found that community diversity and composition are influenced by such activities. Here we combined chemical analysis of water properties and sequencing of fungal internal transcribed spacer regions to explore the relationship between water quality indices and fungal community diversity and composition in three river ecosystem areas along a gradient of anthropogenic disturbance (i.e., less-disturbed mountainous area, wastewater-discharge urban area, and pesticide and fertilizer used agricultural area). Results revealed that the level of anthropogenic activity was strongly correlated to water quality and mycoplankton community. The increase in organic carbon and nitrogen concentrations in water improved the relative abundance of *Schizosaccharomyces*, which could be used as a potential biomarker to reflect pollutant and nutrient discharge. We further applied a biofilm reactor using water from the three areas as influent to investigate the differences in fungal communities in the formed biofilms. Different community compositions were observed among the three areas, with the dominant fungal phyla in the biofilms found to be more sensitive to seasonal effects than those found in water. Finally, we determined whether the fungal community could recover following water quality restoration. Our biofilm reactor assay revealed that the recovery of fungal community would occur but need a long period of time. Thus, this study highlights the importance of preserving the original natural aquatic ecosystem.

## Introduction

Fungi play pivotal roles in the biological processes of many ecosystems (Wong et al., 1998), including as decomposers of river organic matter (e.g., wood and leaf substrates) (Baldy et al., 1995; Pascoal and Cassio, 2004), as parasites or symbionts of algae (Gleason et al., 2008), and as food sources for higher trophic organisms (Kagami et al., 2007). However, fungi are sensitive to environmental change (Hyde et al., 2016). Several studies have shown that environmental variables can affect fungal community composition and function (Chamier, 1987; Artigas et al., 2008; Baudoin et al., 2008; Medeiros et al., 2009; Duarte et al., 2013). For instance, an aquatic survey in south-western France indicated that general distribution patterns of hyphomycete species are associated with altitude and temperature (Chauvet, 1991). Another study on aquatic fungi in relation to the physical and chemical parameters of water quality in Augustow Cana showed that variation in water chemistry can cause significant changes to fungal species diversity (Cudowski et al., 2015). As water quality variation is strongly related to anthropogenic activities (except spatiotemporal factors), many studies have focused on the impacts of such activities on fungal communities. Bergfur and Sundberg (2014) determined that agricultural land use can have significant effects on fungal communities and Pietryczuk et al. (2018) reported that anthropogenically polluted rivers show higher taxonomic diversity of fungi. Overall, most studies on river ecosystems have demonstrated that anthropogenic activities (e.g., nutrient, micropollutant, and microplastic discharge) can shape fungal community composition (Kettner et al., 2017; Tlili et al., 2017; Li et al., 2018).

Due to the close relationship between fungal community variation and anthropogenic activities, using the fungal community as a bioindicator or biomarker is potentially feasible. Previously, hyphomycete communities (richness and composition) have been suggested as integrative indicators for freshwater quality (Pascoal et al., 2005; Sole et al., 2008). To date, however, using the fungal community as a microbial indicator to evaluate anthropogenic impact is still limited.

We hypothesized that fungal community structure may be impacted in response to factors associated with anthropogenic development (Porter-Goff et al., 2010). We selected a typical anthropogenically-disturbed river (Chaobai River) that showed distinct land use partitioning. Integrating high-throughput sequencing for microbial composition determination and flow cells for biofilm cultivation, we correlated environmental variables and fungal community composition in river water and investigated the impact of regional pollution on the characteristics of biofilms. We further evaluated whether contaminated river fungal communities could be restored to their original statuses following river remediation by “indoor remediation” assay. Our study aims to bring scientific knowledge to the river ecosystem evaluation and river management.

## Materials and Methods

### Sampling Sites at Chaobai River

**FIGURE 1:**
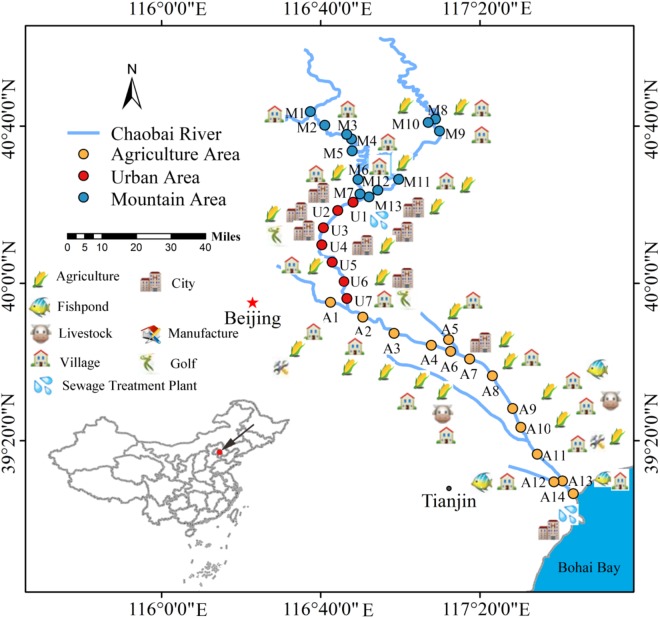
Potential pollution sources and sampling locations along Chaobai River. A total of 34 representative sampling sites were selected and divided into three zones, i.e., mountainous area (MA, blue), urban area (UA, red), and agricultural area (AA, orange). Figure was reproduced and modified with the permission of Liao et al. (2018).

Chaobai River originates from north of Yanshan Mountain in China. It first flows through a less-disturbed mountain area (MA) and then through an urban area (UA) where treated wastewater becomes a main source of river water. Finally, it flows through an agricultural area (AA) before entering the Bohai Bay. The potential pollution sources around Chaobai River are shown in Figure 1. The point-source pollution in the urban area and non-point source pollution in the agricultural area are the main environmental issues facing Chaobai River. According to land use and river length, we selected 34 sampling sites along the river with 13 sites in MA, 7 sites in UA, and 14 sites in AA (Figure 1). Details on Chaobai River are described in our previous study (Liao et al., 2018).

### Water Sampling, Field Measurements, and Chemical Analysis

Sampling campaigns were performed in all four seasons, i.e., December 2016, March 2017, June 2017, and September 2017. Water temperature, conductivity, oxidation–reduction potential (ORP), and pH were recorded at each field site with a multi-parameter water quality sonde (MYRON, Co., United States). Dissolved oxygen (DO) and chlorophyll A (Chl-a) were measured *in situ* with a portable DO meter (HACH, Co., United States) and handheld fluorometer (Turner Designs, United States), respectively.

Surface water (∼250 ml) was loaded into glass bottles with screw caps and acidified to pH < 2 with sulfuric acid for preservation. After transportation to the laboratory, the water samples were filtered through 0.45-μm filter membranes for measurement of soluble reactive phosphorus (SRP), nitrate nitrogen (${\mathrm{NO}}_{3}^{-}$-N), dissolved organic carbon (DOC), and ammonia nitrogen (${\mathrm{NH}}_{4}^{+}$-N) according to standard methods (State Environmental Protection Administration of China, 1989).

### DNA Extraction and Sequencing of ITS1

Surface water (700–900 mL) was filtered using 0.45-μm filter membranes in the field with a pump followed by the addition of LifeGuard^TM^ preservation solution (MoBio Laboratories, Inc., Carlsbad, CA, United States) for DNA sequencing. The filters with preservation solution were transported to the laboratory on ice and stored at -20°C. Total water DNA was extracted from each filter using a PowerWater DNA Isolation Kit (Mo Bio Laboratories, Inc., Carlsbad, CA, United States) according to the manufacturer’s instructions.

The fungal ITS1 gene was amplified using barcoded primers ITS1F (5′-CTTGGTCATTTAGAGGAAGTAA-3′) and ITS1R (5′-GCTGCGTTCTTCATCGATGC-3′) (White et al., 1990; Gardes and Bruns, 1993). The thermal program was set as: 3 min at 95°C, 30 cycles of 30 s at 95°C, 45 s at 55°C, 45 s at 72°C, and finally 10 min at 72°C. The PCR products were then purified with an AxyPrep DNA Gel Extraction Kit (Axygen, United States). The DNA concentration of the purified PCR products was measured by a TBS-380 Fluorometer (Turner BioSystems, Sunnyvale, CA, United States). Sequencing was performed on an Illumina Hiseq 2500 sequencing platform at BGI (Shenzhen, China). Raw sequence reads from 123 samples (13 samples were missing or failed in DNA extraction) were initially filtered to remove low-quality reads and barcode primers. The obtained clean reads were then analyzed using a standard QIIME pipeline (v.1.9.1^1^) (Caporaso et al., 2010). For each sample the two paired-end reads were merged into an ITS1 sequence. The obtained ITS1 sequences were then clustered based on 97% identity and diversity analyses were executed on rarefied data using the core_diversity_analyses.py script in QIIME (Pylro et al., 2014). Taxonomic ranks were assigned to OTU representative sequence using the QIIME/UNITE database as the reference. The pick_open_reference_otus.py script in QIIME was used for the construction of a OTU biom file. The obtained biom file from QIIME was then imported to the ampvis2 (Andersen et al., 2018) and Phyloseq package (McMurdie and Holmes, 2013) in R for diversity analyses. Alpha diversities, including Chao1 and Shannon indices were calculated. Principal coordinate analyses (PCoA) were performed based on Bray–Curtis dissimilarity. Analysis of similarities (ANOSIM) was used to compare the fungal OTUs among groups using the “compare_categories.py” command in QIIME.

To determine the correlations among environmental variables and fungal community composition, we performed detrended correspondence analysis (DCA), with results showing gradient lengths all less than three. This suggested that redundancy analysis (RDA) was a better model to clarify the possible relationship between fungal composition (top 10 abundant genera) and environmental variables. Permutation tests were performed to determine which variables were statistically significant in determining microbial community structure. RDA was conducted using “ggvegan” and the result was plotted using “ggplot2” in R. To ascertain the potential fungal indicator of water quality (e.g., C and N content), we used the TITAN2 package in R (Baker and King, 2010) to clarify sensitive fungal genera influencing variation of C and N concentration in water.

### Biofilm Formation in Flow Cells

Convertible flow cells (Stovall Life Science, Inc., Greensboro, NC, United States), with a dimension of 24 mm × 40 mm × 8 mm, provide continuous culture chambers for the real time, non-destructive study of biofilms under continuous hydrodynamic conditions at a controlled and reproducible flow rate (Supplementary Figure S1). The flow cells were obtained as sterile units and assembled according to the manufacturer’s instructions. The glass layers at the top and bottom of the chamber provide absorbable layers to culture biofilms. The flow cell system was kept free of air bubbles by a bubble trap, which creates low positive pressure on the medium flow, thus mitigating undesirable peristaltic pulsation in liquid delivery to the flow cells. According to the sluggish flow of the Chaobai River^2^, the flow rate in our culturing device was set at a constant rate of 57.33 μl/min using a multichannel peristaltic pump (BT100-1L, Longer Precision Pump, Co., Ltd., Baoding, China) to create favorable conditions for biofilm culturing (Liao et al., 2019).

Biofilms were cultivated for the three groups (MA, UA, and AA) to investigate the impact of regional pollution on the characteristics of biofilms using water collected from the M1, U2, and A8 sites (Figure 1) as influent. Each group had three replicates. Each channel was cultivated for 60 days to collect sufficient biofilms for sequencing. This experiment was conducted twice starting from December 2016 and June 2017, respectively.

To determine whether the contaminated river fungal communities could be restored to their original statuses after river restoration (i.e., point and non-point pollution control), we performed “indoor remediation” in the flow cell reactor as above. Three groups were designed: (i) unpolluted group, whose biofilms were cultured in mountain water for 60 days; (ii) polluted group, whose biofilms were first cultured in mountain water for 20 days, then cultured in urban water for 40 days; and (iii) recovery group, whose biofilms were first cultured in mountain water for 20 days, then cultured in urban water for 20 days, and finally cultured in mountain water for 20 days. Each group was replicated three times in parallel. The experiment was conducted from March 2017.

The continuous-flow cell system for all experiments was incubated at room temperature with natural light. After culturing, the multichannel peristaltic pump was stopped, with a scalpel and pair of forceps then used to collect the biofilms from the glass surfaces of the flow cell system. The small biofilm fragments were collected in a glass beaker. Sterile water (200 mL) was added and stirred evenly to prepare the biofilm suspension. The biofilm suspension was then filtered using 0.45-μm filter membranes. Biofilm DNA was extracted from each filter using a MoBio PowerWater DNA Isolation Kit. The fungal internal transcribed spacer (ITS) gene was amplified and sequenced. The sequence read treatments and statistical analyses were performed as the above description for water DNA.

All statistical analyses were performed using R. All sequence data were deposited in the NCBI Sequence Read Archive database (SRA^3^) (Accession No. SRP125492).

## Results

### Water Quality Along Chaobai River

**FIGURE 2:**
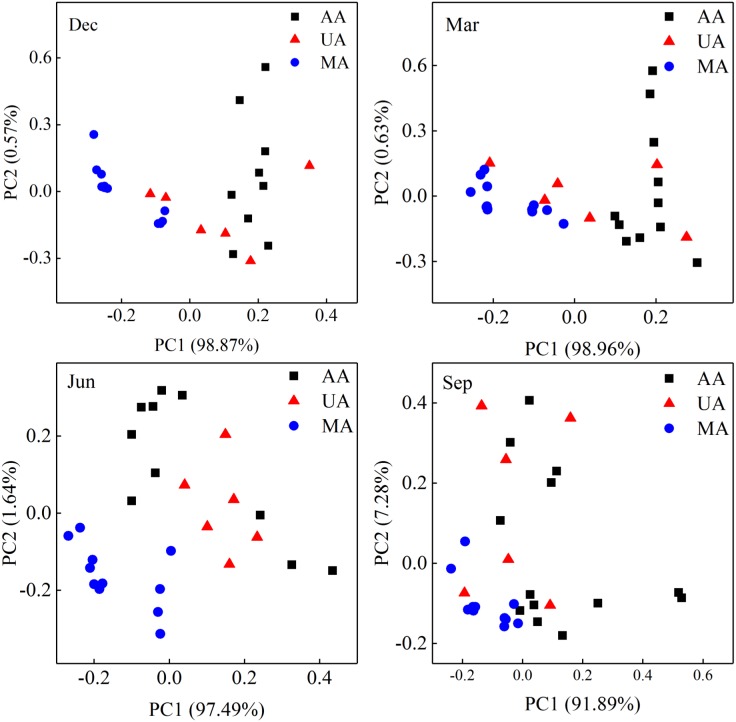
Principal coordinate analyses (PCoA) of water quality over four seasons based on 11 physicochemical indices, including pH, conductivity, ORP, DO, Chl *a*, TN, TP, ${\mathrm{NO}}_{3}^{-}$-N, ${\mathrm{NH}}_{4}^{+}$-N, SRP, and DOC. MA, mountain area; UA, urban area; AA, agricultural area.

The water quality indices in Chaobai River showed obvious differences among the three areas (Supplementary Figure S2). The PCoA results over the four seasons (Figure 2) revealed that the water quality indices demonstrated gradient variation (i.e., dissimilarity in the MA-UA-AA direction) along Chaobai River. Generally, concentrations of TN, ${\mathrm{NH}}_{4}^{+}$-N, ${\mathrm{NO}}_{3}^{-}$-N, TP, Chl *a*, and DOC in MA were comparatively lower than those in UA and AA due to fewer anthropogenic disturbances. UA had a significantly higher level of DOC due to treated wastewater discharge, whereas AA showed higher levels of TP due to fertilizer use (Supplementary Figure S2).

### Planktonic Fungal Community in Chaobai River

**FIGURE 3:**
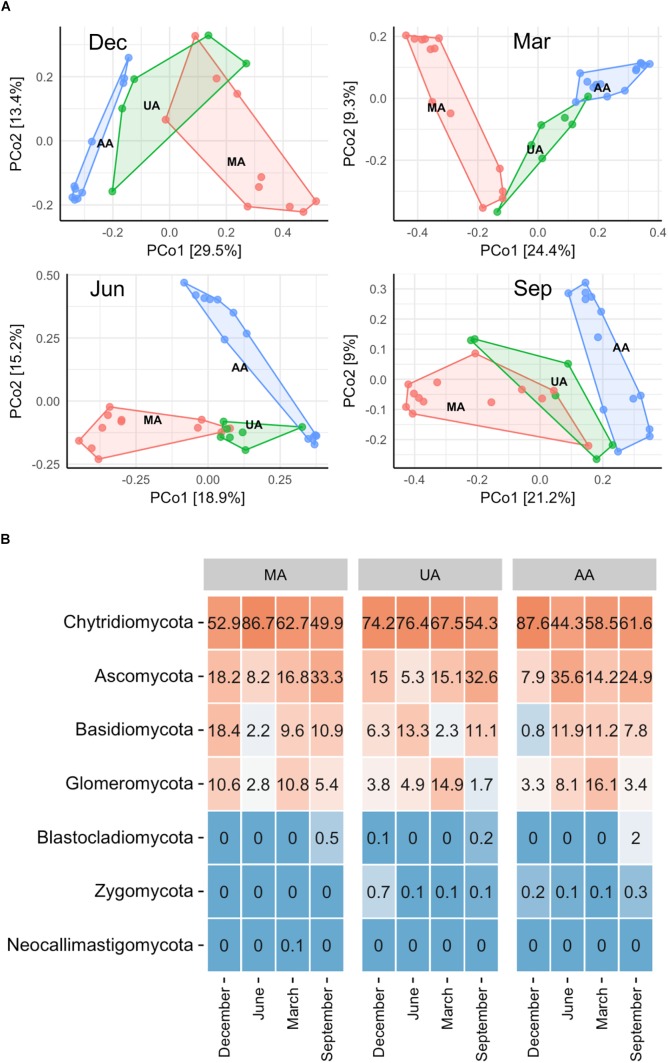
Fungal community distribution among the three areas in Chaobai River across four seasons. **(A)** PCoA of fungal community among the three areas: green, Mountain area; blue, Urban area; red, Agricultural area, **(B)** phylum level community. MA, mountain area; UA, urban area; AA, agricultural area.

To determine fungal compositions in Chaobai River, we performed ITS1 gene sequencing, which produced a total of 7,591,451 qualified reads (14,034–414,425 reads per sample) after quality filtering and removal of ambiguous sequences. Alpha diversity analysis (Supplementary Figure S3) showed that the predicted operational taxonomic units (Chao1) and Shannon diversity indices in MA were all significantly higher (Kruskal–Wallis test, *p* < 0.01) than those in UA and AA. These results suggest that pollutants from treated wastewater and the use of pesticides threaten the survival of some fungi. The PCoA results (Supplementary Figure S4) revealed that the fungal community compositions were significantly affected by water temperature (i.e., season effect t), with low temperature samples (December and March) and high temperature samples (June and September) clustered into two different groups. Hence, to exclude seasonal effects, we analyzed the beta diversity of the fungal community by each season, and found that the fungal communities in AA, UA, and MA still differed but slightly (Figure 3A). Of note, variation showed a gradient (i.e., MA-UA-AA) in accordance with land use (e.g., anthropogenic activity). We used ANOSIM analysis to calculate a constant value *R* (with no difference expressed as 0 and maximum dissimilarity expressed as (1) to determine dissimilarities among groups. Results showed that the three groups exhibited significant differences when seasonal effects were excluded (*R* = 0.65, *p* = 0.01), significantly higher than community composition differences when seasonal effects were included (*R* = 0.14, *p* = 0.01), suggesting the anthropogenic activity was an important factors for altering mycoplankton composition. Accordingly, we compared the relative abundances of identified fungal taxa among the three areas and four sampling seasons. At the phylum level (Figure 3B), we identified seven phyla in total, with Chytridiomycota found to be dominant in all water samples. The relative abundance of each phylum varied with season but not with different area. At the genus level, we identified 425 genera, with *Tuber* abundant in MA, but *Schizosaccharomyces* abundant in UA and AA (Supplementary Figure S5).

### Comparison of Indoor Biofilm Diversity Among the Three Areas

**FIGURE 4:**
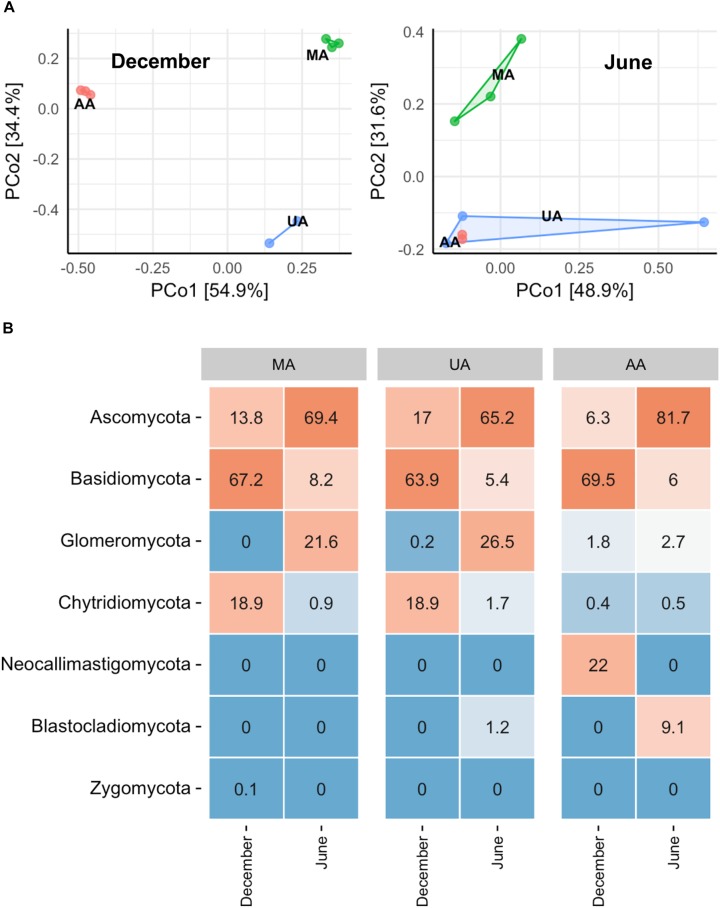
Fungal community composition of reactor biofilms over two seasons of operation. **(A)** PCoA, **(B)** phylum level community. MA, influent using water from mountain area; UA, influent using water from urban area; AA, influent using water from agricultural area.

To investigate whether the MA, UA, and AA biofilms were different, we performed an “indoor biofilm test.” After 60 days of convertible flow cell reactor operation using water from the three areas as influent, we removed the biofilms from the surfaces of the glass layers and extracted DNA. After sequencing, a total of 621,909 high-quality sequences were obtained from 16 biofilm DNA samples, ranging from 2,881 to 84,353 sequences per sample. The Chao1 value of the MA fungal community was significantly higher than that of the UA community (*p* < 0.05) but not than that of the AA community; however, the Shannon indices among the three area biofilms were not significantly different (Supplementary Figure S6). The PCoA analysis based on whole data of two seasons (Supplementary Figure S7) showed the samples between two seasons separated obviously. When analyzing the data by each season, we found that the MA biofilm was significantly different from the UA and AA biofilm samples (Figure 4A). In December, the fungal compositions in the UA and AA biofilms were largely different but in June they were more uniform. Hence, same as for water, seasonal effects were the main driver for biofilm fungal composition, with land use found to be the second most important driver. After analyzing fungal composition at the phylum level (Figure 4B), we observed a clear transition in the dominant phylum from Ascomycota (dominant in December) to Basidiomycota (dominant in June). Comparisons among the biofilms showed that Chytridiomycota was significantly higher in the UA and AA biofilms than in the AA biofilm (*p* < 0.05) in December, whereas Glomeromycota was higher in the UA and AA biofilms in June. At the genus level (Supplementary Figure S8), we found seasonal effects in *Cladosporium* (abundance increasing in June) but did not observe obviously changed dominant genera among the AA, MA, and UA biofilms.

### Fungal Community Alternation in Biofilms With Different Treatment

**FIGURE 5:**
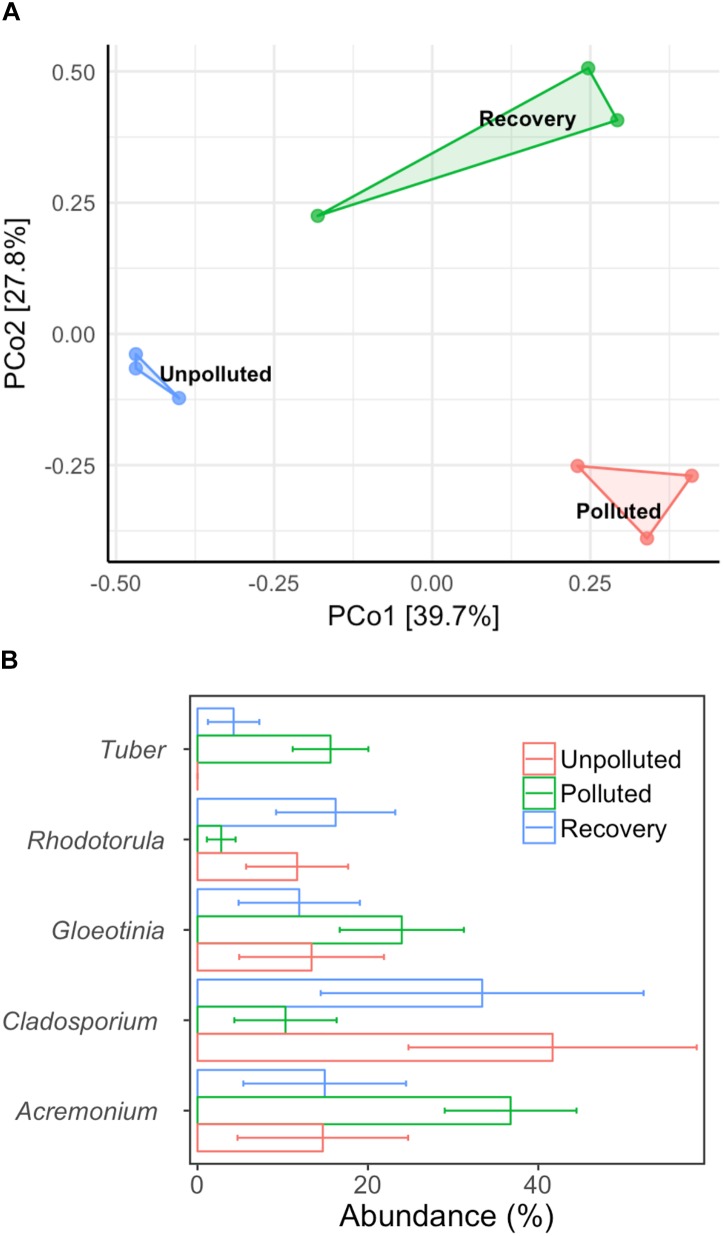
Alternation of biofilm microbial community composition with different treatments. **(A)** PCoA, **(B)** top five dominant genera. Control: mountain water as influent for 60 days; polluted: mountain water as influent for 20 days and urban water as influent for 40 days; recovery: mountain water as influent for 20 days, urban water as influent for 20 days, and finally mountain water as influent for 20 days. Error bar represents mean value plus standard deviation.

We used MA water (unpolluted) and UA water (polluted) as influent in the biofilm reactor and switched the influents at a fixed time to explore the central question of whether the fungal community could recover if water quality was improved from polluted to unpolluted status. After 60 days of operation (running three tests in parallel), the biofilm in each treatment was obtained and DNA was extracted. After sequencing, a total of 140,539 high-quality sequences were obtained from nine biofilm DNA samples, ranging from 7,922 to 22,798 sequences per sample. Unweighted Pair Group Method with Arithmetic mean (UMGMA) cluster analysis showed that the polluted group and recovery group were distinct from unpolluted group (Supplementary Figure S9). From the PCoA results (Figure 5A), we observed a slight recovery transition of fungal community from polluted to unpolluted and ANOSIM analysis verified this as dissimilarity index R switched from 1 (polluted group) to 0.81 (recovered group). Subsequently, we analyzed the five most abundant fungal genera (Figure 5B) and interestingly found that the relative abundances of the dominant genera in the recovery group were similar to those in the unpolluted group but differed from those in the polluted group. The above results suggest that remediation of water quality from polluted to unpolluted status may lead to the recovery of dominant fungi in the biofilm.

## Discussion

Numerous studies have investigated the roles of bacteria in pollutant removal and the nutrient biogeochemical cycle (Bai et al., 2012, 2014), and as microbial indicators for predicting levels of anthropogenic activity and impact (Clements and Rohr, 2009; Garrido et al., 2014) in aquatic ecosystems. In addition to bacteria, fungi also play major roles in the global biogeochemical cycle of nutrients in various aquatic habitats (Jones et al., 2009; Hellal et al., 2016; Song et al., 2018; Tian et al., 2018). In river ecosystems, many studies have indicated that anthropogenic activities (e.g., nutrient and micropollutant discharge) can shape fungal community composition (Tlili et al., 2017; Xiong et al., 2017; Li et al., 2018); however, few studies have used the fungal community as a microbial indicator to evaluate the level of anthropogenic activity and its remediation outcome.

### Correlations Between Anthropogenic Activity and Fungal Community

**FIGURE 6:**
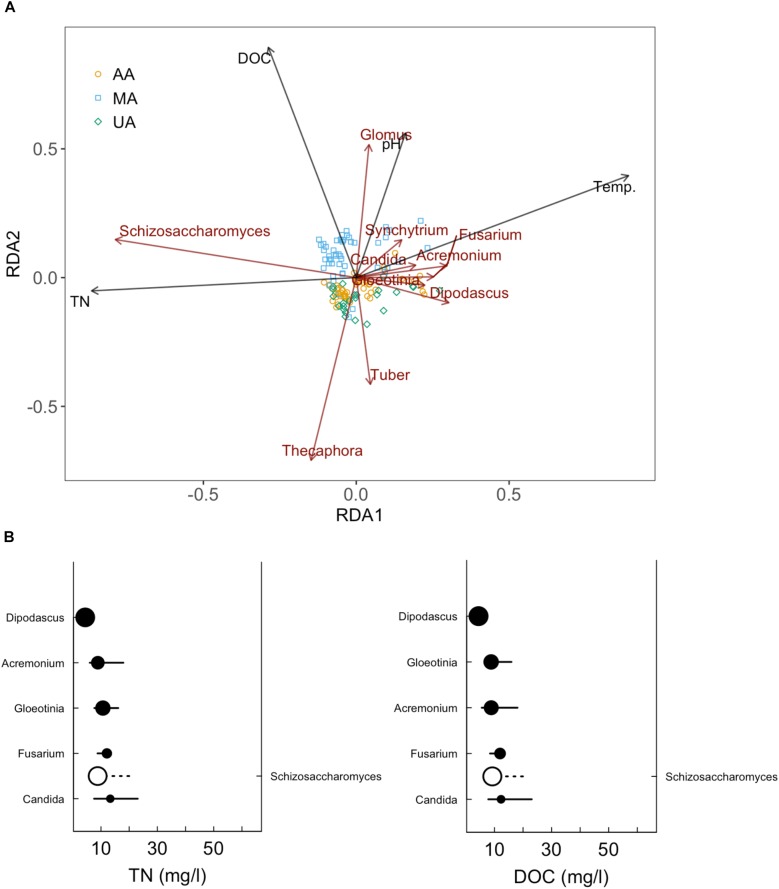
Redundancy analysis (RDA) of fungal community in Chaobai River **(A)** and sensitive genera for TN and DOC increase **(B)**. Top 10 abundant genera were selected for RDA and subsequent sensitive genera analysis.

While fungi are intimately associated with substrates in freshwater systems, the relationship between anthropogenic activities and mycoplankton is less well-defined than that with bacterioplankton (Duarte et al., 2017; LeBrun et al., 2018). Environmental changes, including riparian vegetation changes, physical and chemical disturbances (e.g., pollution), and wet-dry stream cycles, can significantly affect the mycoplankton of rivers (Hyde et al., 2016). As expected, we found that anthropogenic activities (e.g., wastewater discharge and pesticide use) were correlated with declined fungal community diversity and variation of fungal community composition in the river ecosystem, consistent with previous studies (Pascoal et al., 2005; Sole et al., 2008). At the phylum level, we did not observe clear differences among the three areas. Chytridiomycota species, which are ubiquitous and cosmopolitan in aquatic ecosystems (Barr, 1990), were dominant in all three areas (44.3–87.6%). This phylum includes food sources for zooplankton, decomposers of particulate organic matter, parasites of aquatic plants and animals, and converters of inorganics into organics in aquatic ecosystems (Gleason et al., 2008; Rasconi et al., 2011). However at the genus level, the *Tuber* which is affiliated with Ascomycota and is considered as xerophilous fungi (Petr et al., 2012), was abundant in MA, especially in March and June. This genus contains a minimum of 180 species and distributes worldwide (Bonito et al., 2010; Grupe et al., 2018). The high abundance of *Tuber* in MA was probably related to the plant growth in this area. In contrast, *Schizosaccharomyces* (a genus of fission yeasts) was dominant in UA and AA, which was probably due to the discharge of food-related products. After investigating the impacts of anthropogenic activities on the river mycoplankton, we further used mycoplankton as biomarkers to reflect anthropogenic impact. We first correlated water quality indices (e.g., DOC, TN, and TP) and the microbial community to reflect anthropogenic activity levels. The RDA results (Figure 6A) showed that DOC was the critical factor resulting in variance among the three areas. Of the 11 environmental variables, DOC, TN, water temperature, and pH were the dominant factors affecting fungal community composition. Subsequently, we further focused on two indices closely related to anthropogenic activities, and found that *Schizosaccharomyces* was positively correlated with TN and DOC and might outcompete the growth of other fungi in UA and AA (high TN and DOC areas) (Figure 6B). According to the direct relationship between DOC and TN concentrations and the relative abundance of *Schizosaccharomyces* (Supplementary Figure S10), this genus could be potentially used as a biomarker to reflect anthropogenic activities (discharging C and N sources) in Chaobai River.

### Differences Between Water and Biofilm Fungal Community Composition

Biofilms are frequently formed on the surfaces of stone, leaf litter, submerged wood, sediment, and plants within rivers. Biofilm fungal communities can play key roles in the decomposition of submerged organic matter in streams and are important in ecosystem functioning (Pereira et al., 2016). The supply of organic matter and nutrients, as well as biological interactions, can greatly shape biofilm fungal community composition (Suberkropp, 1991; Miura and Urabe, 2017). The nature of the substrata (chemical composition, surface area, stability) colonized by biofilm microbiota has important consequences for biofilm structure and can differentiate biofilm communities among habitats, even within the same environment (Besemer, 2015). Hence, it was difficult to compare the fungal community among the three areas *in situ* due to their different substrata. Thus, we developed an indoor reactor to ensure that all biofilms were formed on the same glass layer substratum. After cultivation, similar to the water fungal community, members of Chytridiomycota and Ascomycota dominated the fungal assemblages, suggesting that both parasitic and saprophytic fungi thrive in biofilms and water (Kettner et al., 2017). However, we also observed clear differences between the water and biofilm fungal community compositions (Supplementary Figure S11), although the biofilm fungi were mainly sourced from the water in the reactor. Furthermore, the biofilm fungi appeared to be more sensitive to temperature as Ascomycota dominated in summer, whereas Basidiomycota dominated in winter. These two phyla are also reported to dominant biofilms of other aquatic ecosystems (Kane et al., 2002; Liu et al., 2015). At the genus level, however, the identified top three fungal genera in the biofilms (Supplementary Figure S8) all belonged to Ascomycota, similar to the water results (Supplementary Figure S5). But unlike water, we did not observe the clear transition of fungal genera between MA and UA/AA, probably due to the homogeneous substrates in three biofilms.

### Can the Fungal Community Recover Following Water Remediation?

Now the Beijing Government has implemented non-point and point pollution control for river ecosystem remediation Thus, it is essential to determine whether these established measures can restore aquatic ecosystems and the time it takes for recovery. Our study clearly demonstrated that when water quality of Chaobai River recovered to “non-polluted” status, however, the biofilm fungal community only established a recovery potential of dominant genera, such as *Tuber*. Microbes, including fungi, are found at the lowest trophic level in the riverine food web. Given bottom-up effects, the recovery of higher trophic organisms in the food web (e.g., invertebrates and vertebrates) would also be slow. Hence, the aquatic ecosystem in Chaobai River could experience long-term recovery after pollution control.

## Conclusion

In this study we explored the response of fungal community to increasing anthropogenic activity in a gradient polluted river. Our research clearly demonstrated that a partition in fungal community composition among the three areas with different human activity gradients. *Schizosaccharomyces* was positively correlated with TN and DOC and thus could be potentially used as a biomarker to reflect anthropogenic activities. Further, our “indoor biofilm test” showed that the biofilms formed from three area water also established a partition on fungal community composition, as same as mycoplankton in the field. Finally, the “indoor biofilm test” revealed the fungal community could be recovered following water quality restoration but would experience much longer time. These results give valuable insights into how anthropogenic activities affect the aquatic fungal community.

## Author Contributions

YB and JQ designed the research. KL, YB, ZJ, and CZ performed the research. YB, QW, and KL analyzed the data. YB and QW wrote the manuscript.

## Conflict of Interest Statement

The authors declare that the research was conducted in the absence of any commercial or financial relationships that could be construed as a potential conflict of interest.

## Footnotes

1 http://qiime.org/

2 http://www.bjwater.gov.cn/bjwater/300817/300821/index.html

3 http://www.ncbi.nlm.nih.gov/Traces/sra/
