# Supplementary material for: Fungal Community as a Bioindicator to Reflect Anthropogenic Activities in a River Ecosystem
Source: Front Microbiol. 2018 Dec 21;9:3152. doi: 10.3389/fmicb.2018.03152 (PMC6308625; doi:10.3389/fmicb.2018.03152)
Supplement: Supplementary file 1 [file Data_Sheet_1.pdf]

# SUPPORTING INFORMATION

## Fungal community as a bioindicator to reflect anthropogenic activities in a river ecosystem

Yaohui Bai [1*], Qiaojuan Wang[1,2], Kailingli Liao [1,2], Zhiyu Jian[1], Chen Zhao[1], Jiuhui Qu[1]

1. Key Laboratory of Drinking Water Science and Technology, Research Center for Eco-Environmental Sciences, Chinese Academy of Sciences, Beijing 100085, China.
2. University of Chinese Academy of Sciences, Beijing 100049, China.

*Corresponding author

Tel: +86 10 62918589

Fax: +86 10 62918589

E-mail: yhbai@rcees.ac.cn

Figure S1. Photo of convertible flow cells reactor.

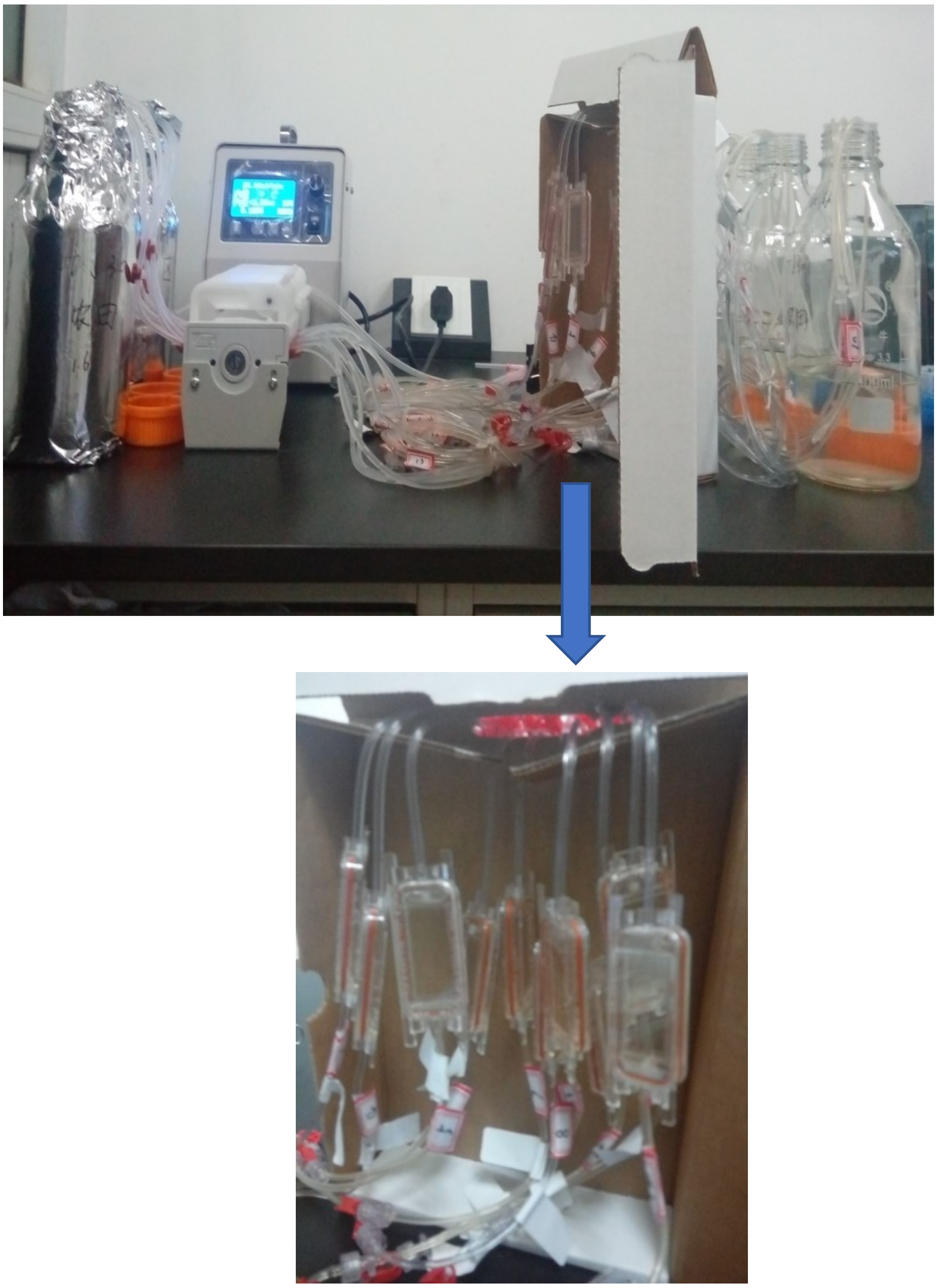

Figure S2. Water properties of MA (Mountain area), UA (Urban area), and AA(Agriculture area) in the four seasons. Asterisks indicate significance level of comparisons: *, significant at $p < 0.05$; **, significant at $p < 0.01$; ***, significant at $p < 0.001$. Numbers along x-axis denote sampling month. Some data were also described in Liao et al. (2018).

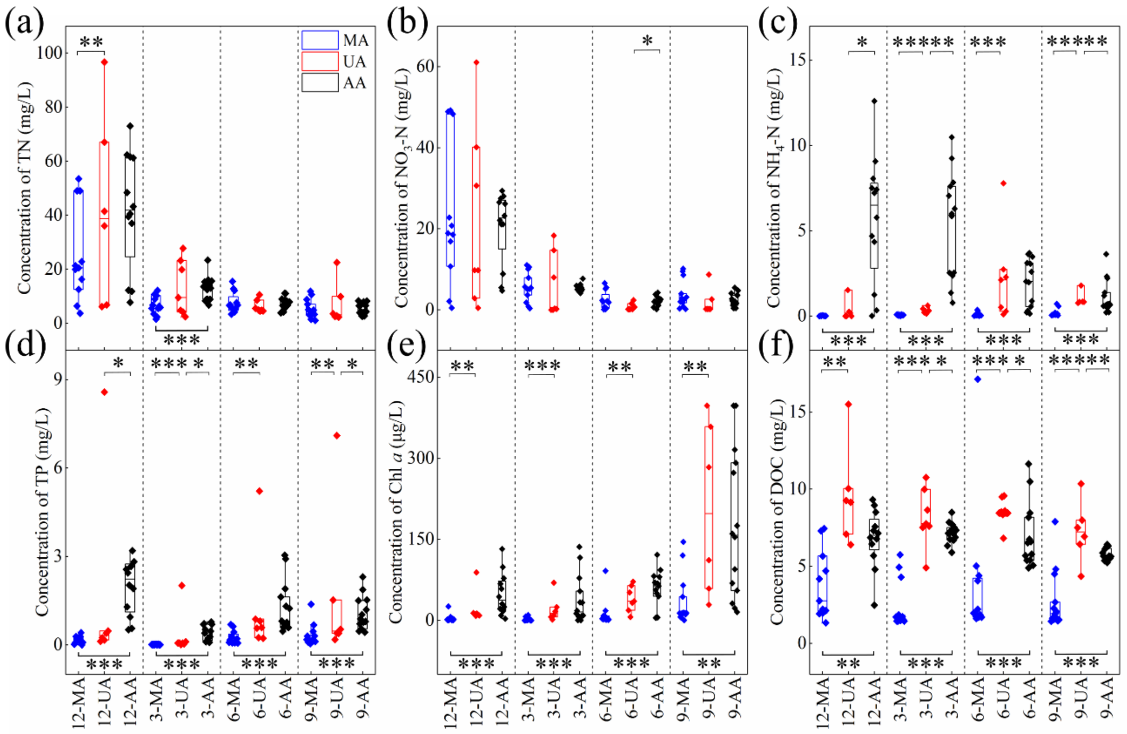

Figure S3. Comparison of alpha diversity of fungal community in the Chaobai River among the three areas. MA: Mountain area, UA: Urban area, AA: Agricultural area. Kruskal-Wallis test was used for comparison among different groups and MA was set as the reference group. ** $p < 0.01$, *** $p < 0.01$, **** $p < 0.0001$. The figure was generated from Phyloseq package (McMurdie and Holmes, 2013) in R.

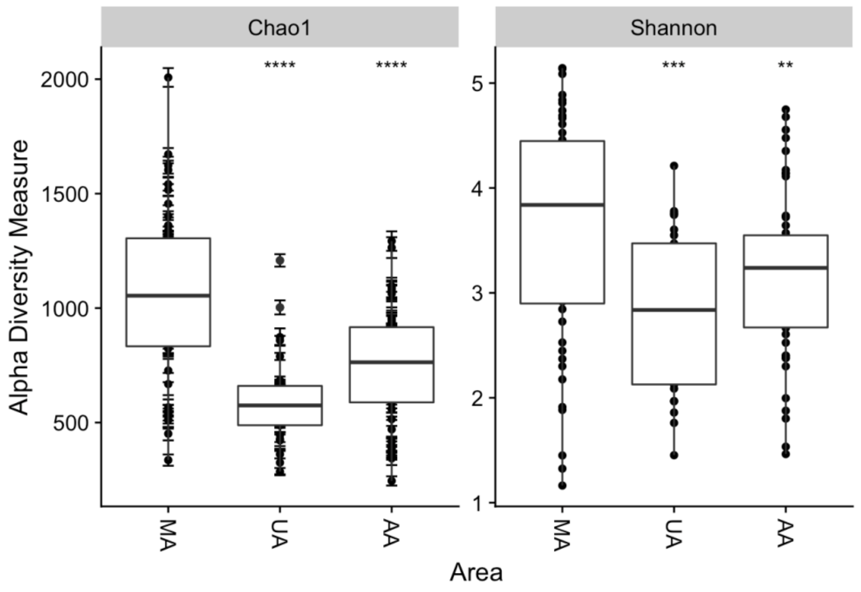

Figure S4. PCoA analysis of fungal community in the Chaobai River. The figure was generated from the ampvis2 package (Andersen et al., 2018) in R.

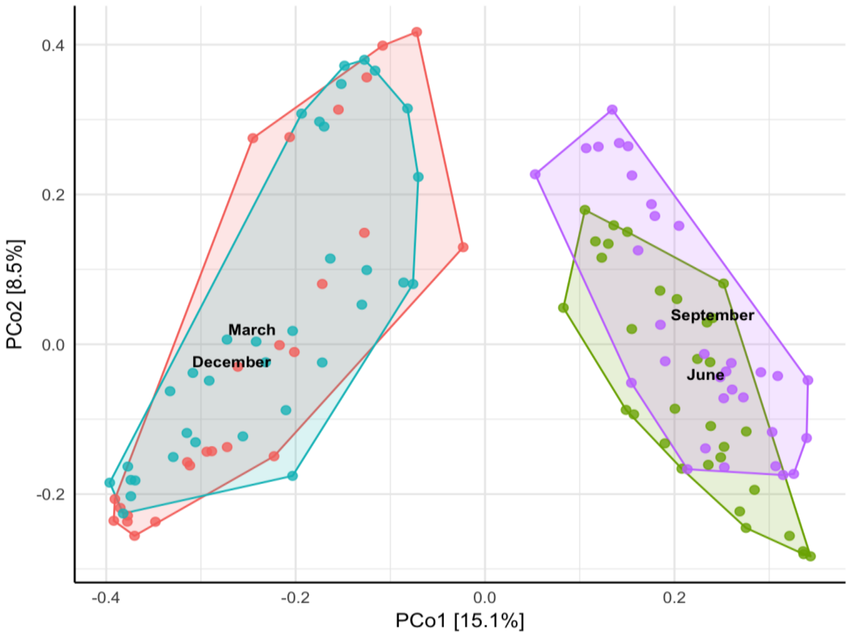

Figure S5. Fungal community composition (top 20 genera) in the water of Chaobai River.

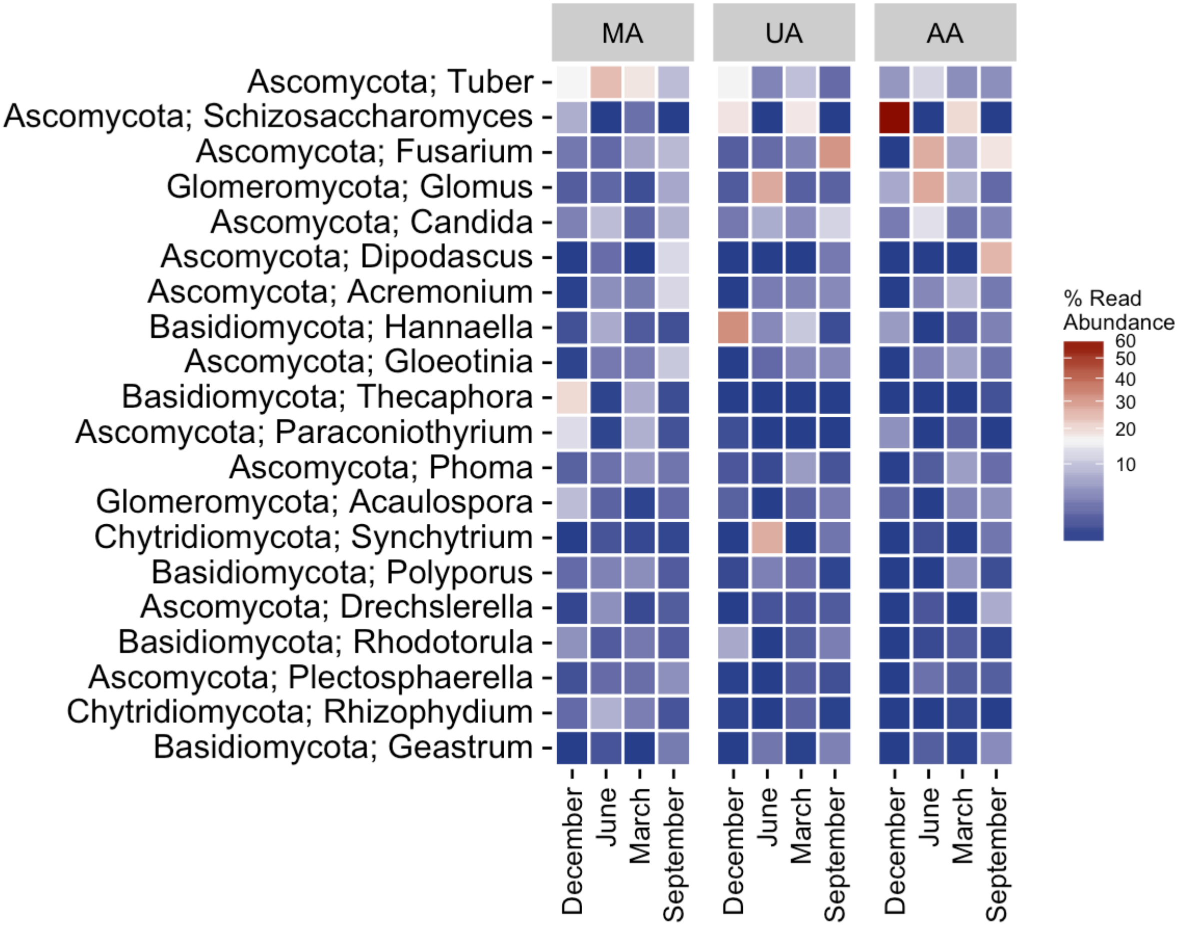

Figure S6. Comparison of alpha diversity of fungal community among the three convertible flow cell reactors using water from three area sites (M1, U2, and A8) as influent. MA: Mountain area, UA: Urban area, AA: Agricultural area. Kruskal-Wallis test was used for comparison among different groups and MA was set as the reference group. * $p < 0.05$; ns: not significant.

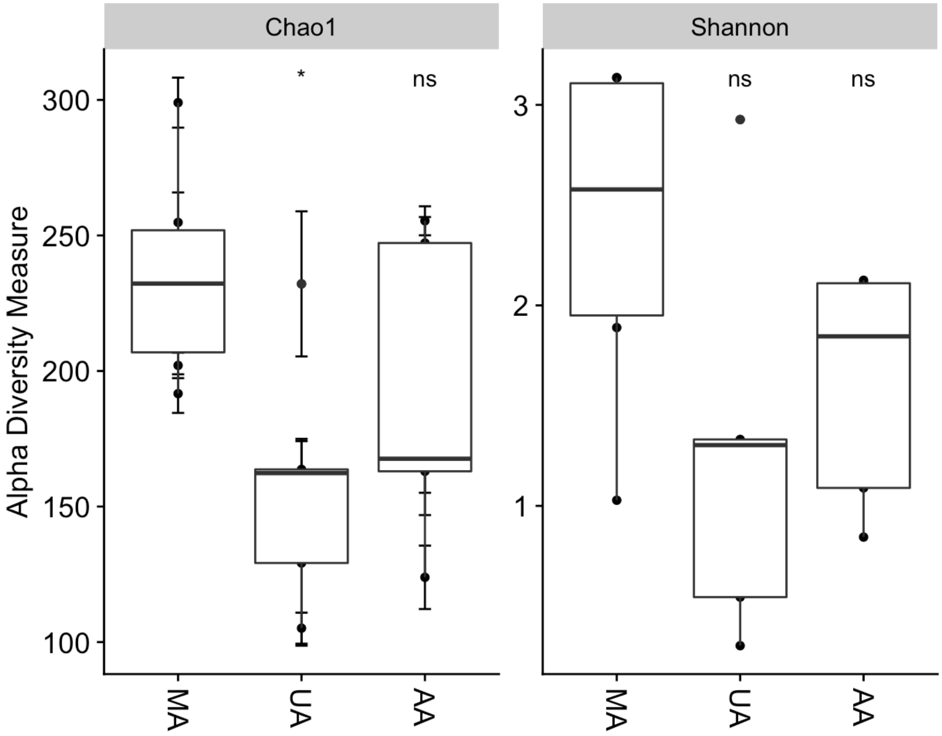

Figure S7. PCoA analysis  of biofilm fungal community

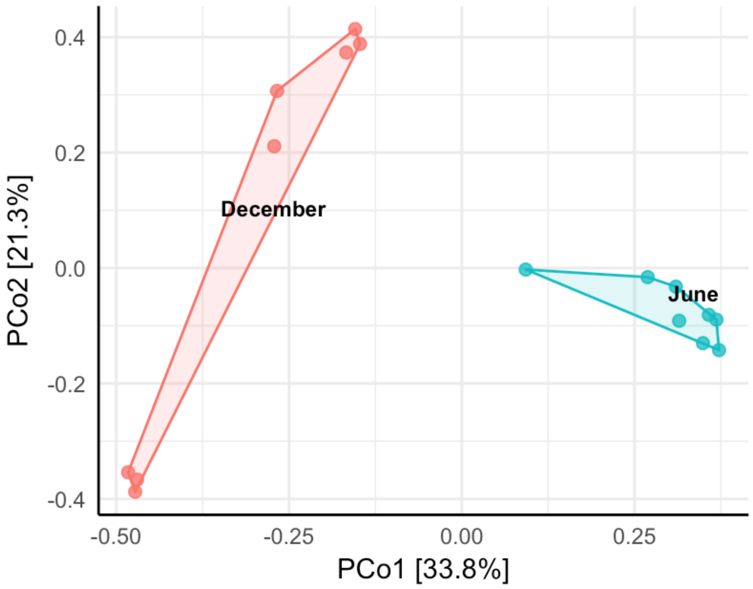

Figure S8. Fungal community (top 20 genera) in the cultivated biofilms with different influents. MA: water from mountain area, UA: water from urban area, AA: water from agricultural area.

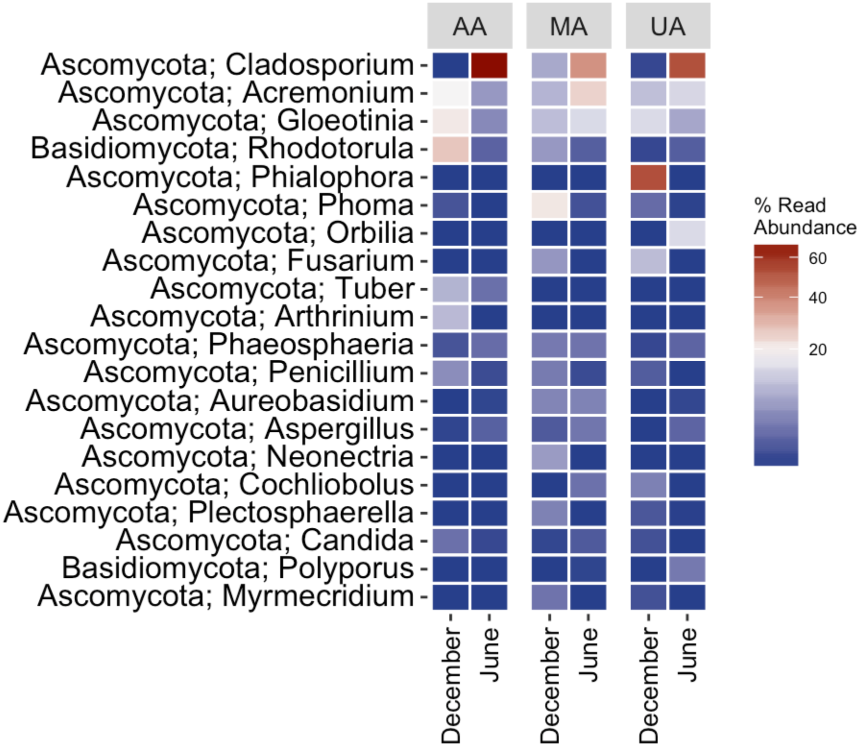


(b)

Figure S9. Unweighted Pair Group Method with Arithmetic mean (UMGMA) cluster analysis of fungal community compositions in three treatments. MC: unpolluted group, MP: polluted group, MR: recovery group. The figure was generated by the software PAST 3.14 (Hammer and Harper, 2006).

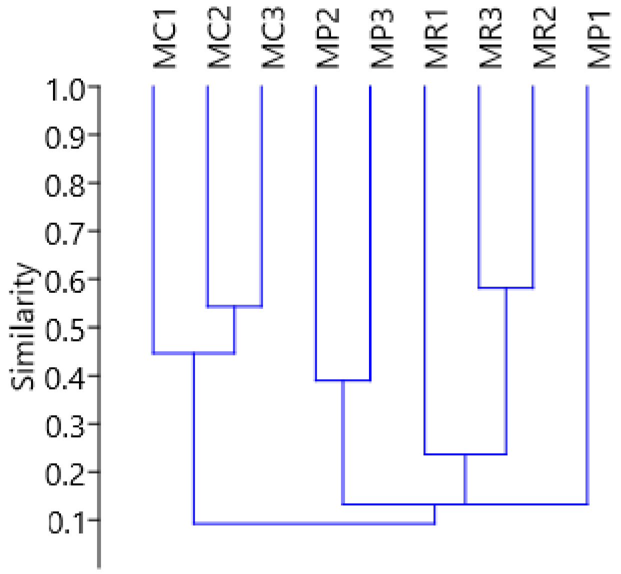

Figure S10. Abundance of *Schizosaccharomyces* along TN and DOC gradient. Overlaid near the top of the plot in red is the observed change point. Overlaid on the abundance plot is the z-weighted probability density function of change point locations across all bootstrap replicates (blue histogram). Figure was plotted using TITAN2 (Baker and King, 2010) package in R.

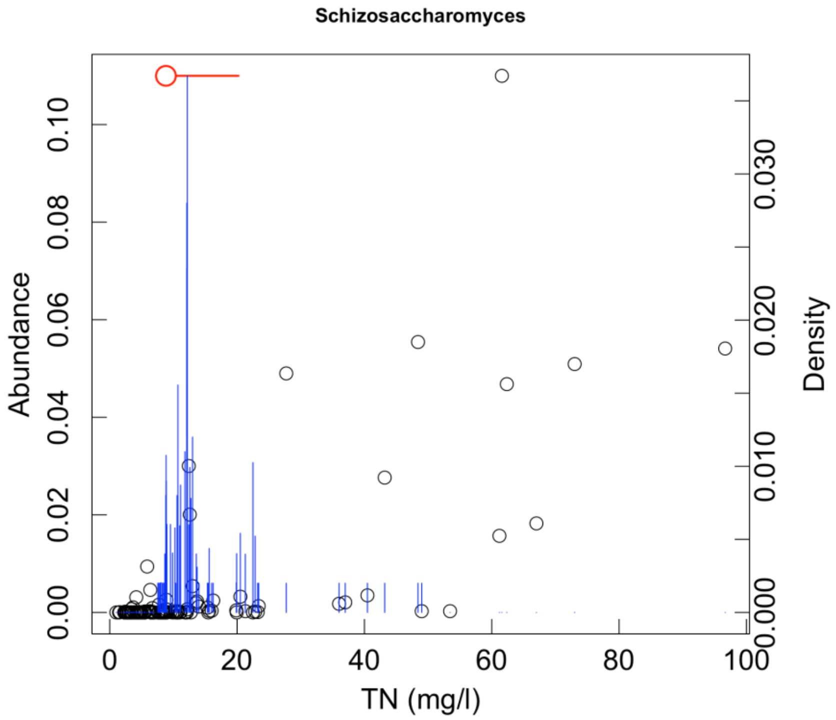


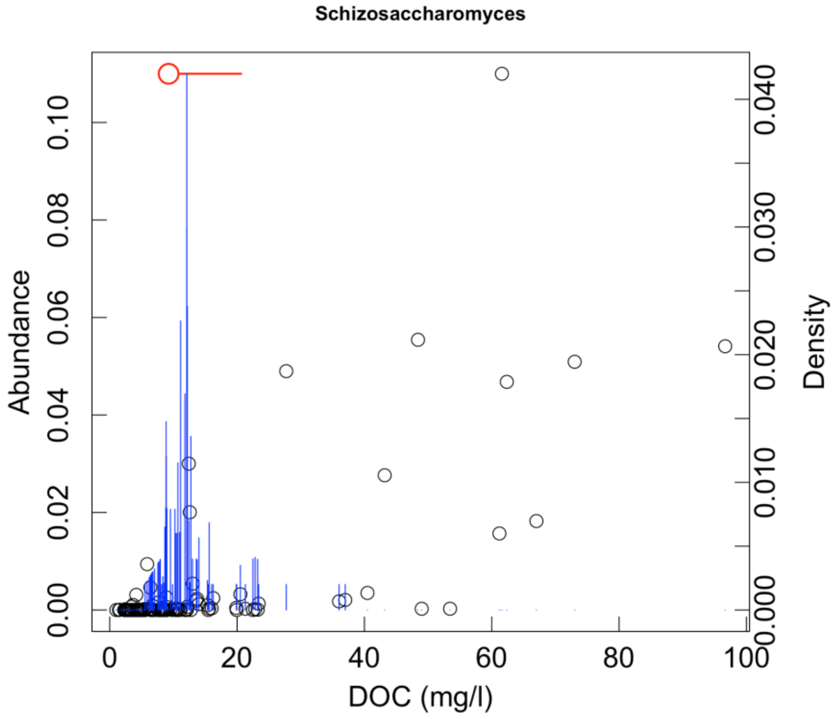

Figure S11. Comparison of fungal community composition between water and biofilm samples (The water samples were only from December and June sampling).

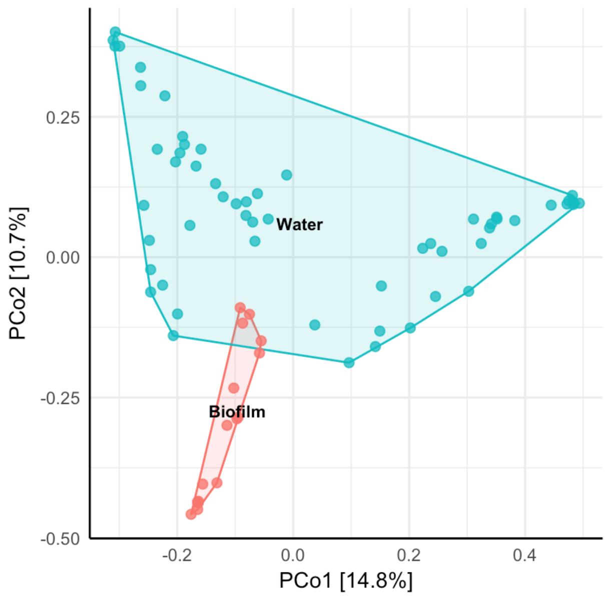

# References

Andersen, K. S. S., Kirkegaard, R. H., Karst, S. M., and Albertsen, M. (2018). ampvis2: an R package to analyse and visualise 16S rRNA amplicon data. *BioBxiv*. https://doi.org/https://doi.org/10.1101/299537

Baker, M. E., and King, R. S. (2010). A new method for detecting and interpreting biodiversity and ecological community thresholds. *Methods in Ecology and Evolution, 1*(1), 25-37. https://doi.org/10.1111/j.2041-210X.2009.00007.x

Hammer, Ø., and Harper, D. A. T. (2006). *Paleontological Data Analysis*: Blackwell.

Liao, K., Bai, Y., Huo, Y., Jian, Z., Hu, W., Zhao, C., et al. (2018). Integrating microbial biomass, composition and function to discern the level of anthropogenic activity in a river ecosystem. *Environ. Int.* 116**,** 147-155. doi: https://doi.org/10.1016/j.envint.2018.04.003.

McMurdie, P. J., and Holmes, S. (2013). phyloseq: An R package for reproducible interactive analysis and graphics of microbiome census data. *PLoS ONE, 8*(4), e61217. https://doi.org/10.1371/journal.pone.0061217
